# Vitamin D_3_ and 25-Hydroxyvitamin D_3_ Content of Retail White Fish and Eggs in Australia

**DOI:** 10.3390/nu9070647

**Published:** 2017-06-22

**Authors:** Eleanor Dunlop, Judy Cunningham, Jill L. Sherriff, Robyn M. Lucas, Heather Greenfield, Jayashree Arcot, Norbert Strobel, Lucinda J. Black

**Affiliations:** 1 School of Public Health, Curtin University, Bentley, WA 6102, Australia; eleanor.dunlop@postgrad.curtin.edu.au (E.D.); j.sherriff@curtin.edu.au (J.L.S.); 2 Food Standards Australia New Zealand (FSANZ), Annerley, Brisbane, QLD 4103, Australia; judyc121@gmail.com; 3 National Centre for Epidemiology and Population Health, Research School of Population Health, The Australian National University, Canberra, ACT 0200, Australia; robyn.lucas@anu.edu.au; 4 Food and Health Research, School of Chemical Engineering, University of New South Wales, Sydney, NSW 2052, Australia; h.greenfield@unsw.edu.au (H.G.); j.arcot@unsw.edu.au (J.A.); 5 National Measurement Institute (NMI), 1/153 Bertie Street, Port Melbourne, VIC 3207, Australia; norbert.strobel@measurement.gov.au

**Keywords:** food composition data, vitamin D_3_, 25-hydroxyvitamin D_3_, fish, eggs

## Abstract

Dietary vitamin D may compensate for inadequate sun exposure; however, there have been few investigations into the vitamin D content of Australian foods. We measured vitamin D_3_ and 25-hydroxyvitamin D_3_ (25(OH)D_3_) in four species of white fish (barramundi, basa, hoki and king dory), and chicken eggs (cage and free-range), purchased from five Australian cities. Samples included local, imported and wild-caught fish, and eggs of varying size from producers with a range of hen stocking densities. Raw and cooked samples were analysed using high performance liquid chromatography with photodiode array. Limits of reporting were 0.2 and 0.1 μg/100 g for vitamin D_3_ and 25(OH)D_3_, respectively. The vitamin D_3_ content of cooked white fish ranged from <0.1 to 2.3 μg/100 g, and the 25(OH)D_3_ content ranged from 0.3 to 0.7 μg/100 g. The vitamin D_3_ content of cooked cage eggs ranged from 0.4 to 0.8 μg/100 g, and the 25(OH)D_3_ content ranged from 0.4 to 1.2 μg/100 g. The vitamin D_3_ content of cooked free-range eggs ranged from 0.3 to 2.2 μg/100 g, and the 25(OH)D_3_ content ranged from 0.5 to 0.8 μg/100 g. If, as has been suggested, 25(OH)D_3_ has five times greater bioactivity than vitamin D_3_, one cooked serve (100 g) of white fish, and one cooked serve of cage or free-range eggs (120 g) may provide 50% or 100%, respectively, of the current guidelines for the adequate intake of vitamin D (5 µg) for Australians aged 1–50 years.

## 1. Introduction

The 2011–2013 Australian Health Survey revealed that 23% of Australian adults were vitamin D deficient (serum 25-hydroxyvitamin D (25(OH)D) concentrations <50 nmol/L) [1]. Sunlight exposure offers the greatest potential source of vitamin D via the endogenous, two-step process of ultraviolet-B (UVB) photolytic and thermal conversion of cutaneous 7-dehydrocholesterol to produce vitamin D_3_ [2]. However, the effectiveness of sun exposure as a source of vitamin D_3_ varies with geographical location, season, skin pigmentation, lifestyle and environmental factors [3]. Furthermore, sunlight exposure is often limited due to sun-safety concerns. As such, the maintenance of vitamin D sufficiency in some people relies on dietary vitamin D, which is found naturally in few foods and generally in low concentrations. Fish, meat, eggs and dairy are sources of vitamin D_3_, while vitamin D_2_ is found in mushrooms. The hydroxylated form of vitamin D_3_, 25(OH)D_3_, is also obtained from animal sources, and may be up to five times more bioactive than vitamin D_3_ [4,5]. It is recognised that omitting the 25(OH)D_3_ content of animal products from food composition databases is very likely to lead to underestimates of true vitamin D intake [6]. Hence, the accurate determination of the total vitamin D content of fish, meat, eggs and dairy requires measurement of both vitamin D_3_ and 25(OH)D_3_.

In Australia, dietary correction of vitamin D deficiency is complicated by lack of locally relevant vitamin D food composition data [7]. Since variations in nutritional content of produce may occur with geographical location, climate, feed fortification and animal production practices [8], it is inappropriate to rely on international vitamin D composition data. Furthermore, international data may not represent species and types of foods commonly consumed in Australia [9]. Since 2013, vitamin D_3_ and 25(OH)D_3_ have been measured in a limited number of Australian meat [10], egg [8] and seafood [11] samples, based on convenience or purposive sampling. We build on these studies by presenting new data for the vitamin D_3_ and 25(OH)D_3_ content of white fish and eggs purchased from retail outlets across five cities in Australia.

## 2. Materials and Methods

Sample purchasing, preparation and analysis were carried out by the National Measurement Institute of Australia (NMI), Port Melbourne, Victoria, which is accredited by the National Association of Testing Authorities for analysing vitamin D in foods (Accreditation No.: 89).

### 2.1. Sample Purchasing

A single-species fish sample (2 kg) was purchased in each of five Australian cities: Adelaide, Brisbane, Melbourne, Perth and Sydney. Species were selected based on those most commonly reported to be consumed in the 2011–2013 Australian Health Survey [12]. Samples of hoki (*Macruronus* spp.), king dory (*Cyttus traversi*), basa (Pangasiidae) and barramundi (*Lates calcarifer*) were purchased. Fresh, skinless fillets were purchased in preference to frozen or skin-on fillets to simplify sample preparation and reflect usual preparation practices. Frozen, crumbed, marinated fish or oily fish were excluded. Fish samples were purchased from an independent fish shop in Melbourne, from an independent supermarket or fish shop in Sydney, and from one of three different major supermarket chains in Perth, Adelaide and Brisbane. Point-of-sale packaging labels were photographed to record species name, country of origin and, if specified, whether the fish was wild-caught or farmed. Retail sources, origins and quantities of fish samples are shown in Table 1.

Ten dozen chicken eggs were purchased in each of five Australian cities (Adelaide, Brisbane, Melbourne, Perth and Sydney), five labelled as cage and five labelled as free-range. Eggs labelled as “free to roam”, “eco” or “barn-laid” were excluded. In Melbourne, eggs were requested from either an independent grocery shop, farmers’ market or boutique outlet. In Sydney, eggs were sourced from an independent supermarket. In Perth, Adelaide and Brisbane, eggs were purchased from one of three different major supermarket chains. In order to include eggs of different sizes, large eggs (600 g per dozen) were prioritised for purchase in Melbourne and Adelaide, with extra-large eggs (700 g per dozen) to be purchased only if large eggs were unavailable. Extra-large eggs were prioritised for purchase in Sydney, Perth and Brisbane, with large eggs to be sourced only if extra-large eggs were unavailable. Egg packages were photographed to identify brand, minimum package weight and type. Purchase and “best before” dates were recorded. The brands purchased were Lodge Farms, Swan Valley Egg Company, Elderbrook, McLaren Vale, Pioneer, Willow Zen and Pirovic. Retail sources, origins and quantities of egg samples are shown in Table 1.

### 2.2. Sample Preparation and Analysis

**Figure 1:**
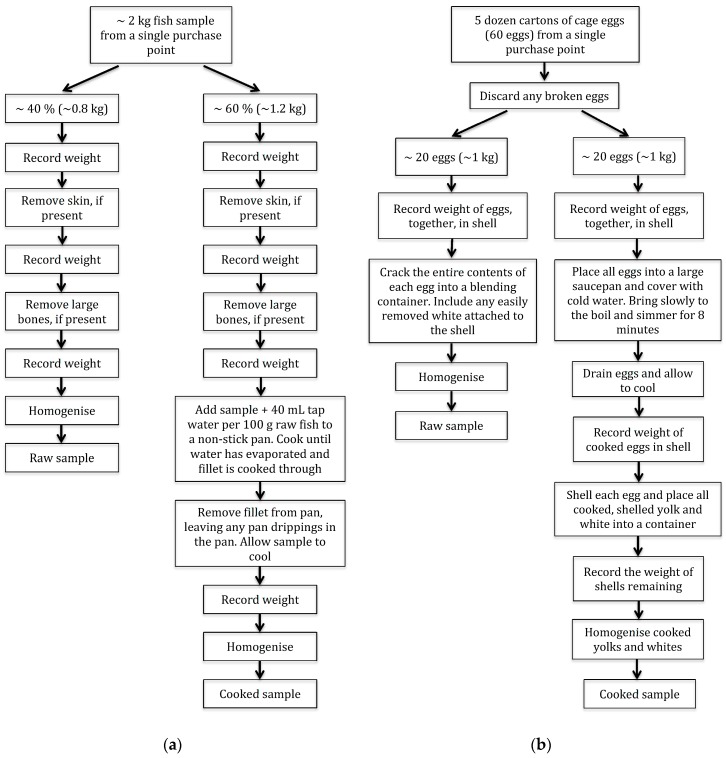
Preparation of (**a**) fish and (**b**) cage eggs for analysis of vitamin D_3_ and 25-hydroxyvitamin D_3_. Free-range eggs were prepared as per cage eggs.

All fish and egg samples were chilled at 3 °C from time of purchase to preparation; samples were protected from air and light by original packaging, tight foil wrapping or sealed containers. Upon arrival at the laboratory, samples were prepared as soon as possible to minimise sample and vitamin D_3_/25(OH)D_3_ deterioration. Each of the five fish samples were prepared and cooked according to the process shown in Figure 1a, yielding one raw and one cooked analytical sample for each of the five purchased samples. Eggs were prepared and hard-boiled according to the process shown in Figure 1b, yielding one raw and one cooked analytical sample for cage eggs, and one raw and one cooked analytical sample for free-range eggs, for each of the five purchase locations. Following preparation, samples were frozen at −18 °C and protected from light and oxygen until analysis.

Vitamin D_3_ and 25(OH)D_3_ were measured by high performance liquid chromatography with photodiode array (HPLC PDA) [14]. Quality control procedures included an in-house control (infant formula) and a spiked sample of barramundi. All samples were analysed in duplicate and the relative percent differences between duplicate analyses were recorded. For total fat, the method of analysis was VL302: Fat Determination in Food and Biota by Mojonnier Extraction [15]. Moisture determination (VL298) (in-house method based on published methodology [16]) was made-according to sample matrix type, using either sand and vacuum drying, or no sand and conventional drying. Fish species identity was confirmed in singlicate by DNA profiling (restriction fragment length polymorphism-polymerase chain reaction) [17].

### 2.3. Quantifying Vitamin D Equivalents

For fish, duplicate values for each purchased sample were averaged (separately for raw and cooked samples). Duplicate values for each purchased sample of cage eggs and free-range eggs were averaged (separately for raw and cooked samples). For eggs, the average values were then averaged again across the five purchased samples to give one mean value of vitamin D_3_ and 25(OH)D_3_ for cage eggs (raw and cooked) and one mean value of vitamin D_3_ and 25(OH)D_3_ for free-range eggs (raw and cooked). For the purpose of this article, we use the term “vitamin D equivalents” (VitDE) to denote the total vitamin D content of foods as follows: VitDE = vitamin D_3_ + (5 × 25(OH)D_3_) [4,7]. True retention percentage of VitDE in cooked food was calculated as follows: (VitDE per g cooked food × g of cooked food)/(VitDE per g raw food × g of raw food) × 100.

### 2.4. International Vitamin D Composition Data

We searched key international databases, including the United Kingdom’s McCance and Widdowson’s The Composition of Foods [18], the US Department of Agriculture National Nutrient Database for Standard Reference (Release 28, released September 2015, slightly revised May 2016) [19] and Food EXplorer [20] for vitamin D_3_ and 25(OH)D_3_ composition data for white fish and eggs. Only analytical values with identified method and data sources were gathered—values estimated from related foods were excluded. Values were gathered only where it was clear whether the sample was raw or cooked. Where cooking method was specified, hard-boiled was selected for eggs; poached and fried eggs were omitted.

## 3. Results

### 3.1. Quality Control Results

The limits of reporting (LOR) were 0.2 and 0.1 µg/100 g for vitamin D_3_ and 25-hydroxyvitamin D_3_, respectively. The recovery from the assigned value for vitamin D_3_ in the in-house control (infant formula) was 97%, with the relative percentage difference between replicates averaging 9%. The recovery from a spiked sample of barramundi was 86% for vitamin D_3_ and 87% for 25-hydroxyvitamin D_3_. Across all fish and egg samples, raw and cooked, the relative percentage difference between duplicate samples for moisture, fat, vitamin D_3_ and 25-hydroxyvitamin D_3_ was 0.1%, 2.4%, 8.7% and 11.1%, respectively.

### 3.2. White Fish

Since fish samples were purchased without packaging, use-by dates were not specified: retail signs informed of fish type, country of origin and price per kilogram, whilst packaging and labelling added at the point-of-sale included the type, net weight, price per kilogram, total price and purchase date. Retailers did not always specify whether fish were farmed or wild-caught. DNA profiling of fish samples confirmed correct species labelling. Systematic names were obtained from the Australian Fish Names Standard Database [21].

Values for moisture, fat, vitamin D_3_ and 25(OH)D_3_ in white fish are reported in Table 2, together with retention percentages of VitDE for cooked samples. With the exception of basa (Pangasiidae) purchased in Perth, all samples contained concentrations of vitamin D_3_ greater than the LOR. All samples contained 25(OH)D_3_ in concentrations greater than the LOR. In raw white fish, the mean VitDE content was 2.94 μg/100 g wet weight (range, 1.50–5.80 μg/100 g). The mean VitDE in cooked fish was 2.48 μg/100 g wet weight (range, 2.15–3.00 μg/100 g). Barramundi (*Lates calcarifer*) contained the most VitDE of raw fish analysed in this study with 60% contributed by 25(OH)D_3_. In cooked samples, VitDE was highest in basa with the entire amount attributed to 25(OH)D_3_. Barramundi retained the least vitamin D upon cooking (35%), whereas basa retained the most (163%). VitDE retention in hoki (*Macruronus* spp.) varied widely, from 58% to 119%, with respective variations in retention of vitamin D_3_ and 25(OH)D_3_ of 40–354% and 60–103%. All fish samples contained fat levels equal to, or less than, 3 g/100 g (wet weight).

### 3.3. Eggs

Best before dates for eggs ranged from 20 to 37 days post-purchase date. Although large eggs (600 g per dozen) were preferred for Melbourne, free-range eggs were only available as extra-large (800 g per dozen) from the retailer in Melbourne. For free-range eggs, stocking densities ranged from 130 to 10,000 hens per hectare.

Values for moisture, fat, vitamin D_3_ and 25(OH)D_3_ in eggs are reported in Table 3, together with retention percentages of VitDE for cooked samples. Overall, average VitDE content did not vary markedly between cage and free-range eggs; however, 25(OH)D_3_ contributed 93% of VitDE content in cage eggs, and 73% in free-range eggs. Considerable variation in VitDE was seen in samples from different cities, with VitDE for raw cage eggs ranging from 3.6 μg/100 g in Perth to 7.0 μg/100 g in Melbourne and Adelaide, and VitDE for raw free-range eggs ranging from 3.1 μg/100 g in Perth to 9.0 μg/100 g in Brisbane. A greater than 5.5-fold difference existed for vitamin D_3_ in raw, free-range eggs from Adelaide (1.7 μg/100 g) compared to those purchased in Sydney (0.3 μg/100 g). VitDE retention upon cooking also varied greatly between cities, ranging from 39–104% in free-range eggs and 36–113% in cage eggs.

### 3.4. International Vitamin D Composition Data

Internationally sourced data for white fish and eggs are presented in Table 4 and Table 5, respectively. The most recent UK analyses measured 25(OH)D_3_ by HPLC; however, the vitamin D_3_ and 25(OH)D_3_ content of white fish were recorded as either trace or unreported [18]. In the USA, HPLC or HPLC-LC/MS were used to measure vitamin D_3_ in white fish [22]; however, 25(OH)D_3_ was not measured as the analytical methodology used to determine this metabolite of vitamin D was considered to be insufficiently validated at that time. Even without considering 25(OH)D_3_, VitDE in raw and cooked halibut (*Hippoglossus hippoglossus* (L.) and *H. stenolepis* (Schmidt)) in the USA were relatively high due to the high vitamin D_3_ content. Both vitamin D_3_ and 25(OH)D_3_ were measured in chicken eggs in the UK. In The Netherlands, egg analyses were carried out by HPLC, with values for vitamin D_3_ and 25(OH)D_3_ combined and reported as total vitamin D, without use of a bioactivity factor [23].

## 4. Discussion

In our study, the vitamin D_3_ and 25(OH)D_3_ content varied widely between fish samples, which could reflect variation in production practices, feed type and differences within species that may occur due to location, season, and water clarity [26,27]. Barramundi (*Lates calcarifer*) contained the highest VitDE of the species analysed in our study, although the content was less than in Australian farmed barramundi (10.7 μg/100 g) analysed by LC-IT-MS in 2014 by Padula and colleagues [11]. The farmed barramundi sample analysed by Padula and colleagues was sourced directly from the point of production, which may have minimised any vitamin D or 25(OH)D_3_ deterioration through storage, whilst fortification of farmed fish feed or aforementioned variations in location or time of year may also have contributed to the higher VitDE in that study [11]. In our study, samples of king dory (Melbourne), hoki (Adelaide) and basa (Perth) showed higher fat and VitDE content (per 100 g) after cooking, compared with raw. This outcome has also been demonstrated in pork, and can be attributed to moisture loss [28]. VitDE in white fish in our study was greater than in international samples, with the exception of the halibut analysed in the USA. While the same environmental, geographical and production effects that cause variation within Australian samples may play a role, sensitivity of the analysis method and accurate determination of 25(OH)D_3_ may also result in substantial differences in VitDE.

We found no overall difference in VitDE between free-range and cage eggs; however, considerable variation was seen in samples from different cities. Vitamin D content may vary in eggs according to feed fortification and exposure of hens to UV-B radiation [29,30]. Layer feed fortification with vitamin D_3_ is common practice in Australia with usual amounts of approximately 75 μg of vitamin D_3_ per kilogram of feed [31]. Kühn and colleagues found that free-range farming was effective in raising the vitamin D content of eggs, but only when hens had adequate access to suitable outdoor areas [30]. This finding was reflected in a recently published study that showed greater vitamin D_3_ concentrations in both free-range and organic egg yolks, and higher 25(OH)D_3_ content in organic egg yolk, compared with egg yolks from hens kept indoors [32]. Authors suggested that sunlight exposure was a likely influence. A previous study in Australia found that egg yolk contained 38% more vitamin D_3_ and 300% more 25(OH)D_3_ when hen feed was fortified with both vitamin D_3_ and 25OH)D_3_ [8]. Feed fortification with only vitamin D_3_ also increases the content of both vitamin D_3_ and 25(OH)D_3_ in eggs [33]. Our study included free-range eggs from a low-stocking density farm (130 hens/ha)—these eggs had higher vitamin D_3_ (but not 25(OH)D_3_) content than eggs from farms with higher stocking densities. Further research, involving sampling eggs from farms using various stocking densities where feed composition is known, would be required to investigate whether any differences in the vitamin D_3_ and 25(OH)D_3_ content of eggs is due to production practices (e.g., sunlight exposure) or feed fortification.

In international data sources, the vitamin D_3_ content of eggs was generally higher than in our Australian samples. However, since the 25(OH)D_3_ content of international samples was either very low (UK), unreported (USA) or not adjusted for bioactivity (The Netherlands), the considerably higher amounts of 25(OH)D_3_ detected in our egg samples led to greater VitDE content overall compared with international data.

The adequate intake (AI) of vitamin D for Australians aged 1–50 years is 5 μg/day, increasing to 10 μg/day for 51–70 year olds, and 15 μg/day for those aged >70 years [34]. If, as has been suggested, 25(OH)D_3_ has five times greater bioactivity than vitamin D_3_, one cooked serve (100 g cooked weight) of white fish may supply between 43% and 60% of the AI for 1–50 year olds (depending on the fish species), and one cooked serve (2 large eggs, 120 g) of eggs may provide 100% of the AI for 1–50 year olds. However, it has been acknowledged that the current guidelines for the AI of vitamin D in Australia are out of date [35]. In the United States, the recommended dietary allowance for vitamin D is 15 µg/day for infants, children and adults aged ≤70 years (including during pregnancy and lactation) and 20 µg/day for adults aged >70 years [36]. The European Food Safety Authority recently set an AI for vitamin D at 10 μg/day for infants aged 7–11 months and 15 μg/day for children aged 1–17 years and adults [37].

Both white fish and eggs available in Australia may provide nutritionally useful amounts of vitamin D; however, conveying this information to consumers is hampered by the lack of Australian food composition data. In addition, 25(OH)D_3_ is not recognised in the Australia New Zealand Food Standards Code as contributing to the vitamin D content of foods [38], despite 25(OH)D_3_ being used as a fortificant in animal feed, including in layer poultry farming [8]. The Food Standards Code states that one serve of a food must contain at least 1 µg of vitamin D for a general “source of” claim, or 2.5 µg of vitamin D for a “good source of” claim [38]. As our study demonstrates, 25(OH)D_3_ may contribute substantially to the vitamin D content of foods. Although the potency of 25(OH)D_3_ compared with vitamin D_3_ is debated, there is general agreement that 25(OH)D_3_ is more bioactive than vitamin D_3_ [4,39,40,41,42,43]. Therefore, failure to measure 25(OH)D_3_ in food may result in considerable underestimation of vitamin D intakes. According to Heaney and colleagues, the finding of 25(OH)D_3_ in food provides a possible explanation for the gap between the calculated total basal input of vitamin D (sun exposure plus traditional food sources of vitamin D) and measured serum 25(OH)D concentrations [44].

A major strength of our study was the measurement of 25(OH)D_3_, which is frequently lacking in food composition databases. Accurate determination of 25(OH)D_3_ at low levels is crucial, since each amount unmeasured may represent up to a five-fold loss in overall vitamin D content, leading to underestimation of intake and misrepresentation of intake versus requirement. A further strength of this study was the multi-city sampling plan: we sampled from five major Australian cities, which reflect where the majority of Australians are buying food. Financial constraints meant that we were only able to explore a limited selection of white fish species available in Australia. We did not explore the differences between skin-on versus skin-off fish fillets, nor did we compare different cooking methods for fish and eggs. A comparison of wild versus Australian farmed fish may be warranted, since Lu and colleagues showed that farmed salmon in the United States had approximately 25% of the vitamin D content of wild salmon [45]. Egg yolk and white were not analysed separately, nor were dried versions of any form. All samples were purchased in March, at the end of the Australian summer; hence, the effect of seasonality was not investigated.

## 5. Conclusions

This study demonstrates that significant amounts of 25(OH)D_3_ are present in white fish and eggs, and is a preliminary step in updating the vitamin D composition data for Australian foods. Our results demonstrate the importance of having Australia-specific vitamin D composition data since, at least for white fish and eggs, the vitamin D content of foods available in Australia differs from foods available internationally, and different fish species are available in Australia versus internationally. White fish and eggs in Australia contain amounts of vitamin D_3_ and 25(OH)D_3_ that may assist people in achieving the AI for vitamin D. As a geographically large and climatically diverse country, Australia requires representative, wide-ranging sampling of local produce to accurately quantify vitamin D intakes in the population, and to determine whether future food-based strategies to correct vitamin D deficiency are warranted, safe and effective.

## Figures and Tables

**Table 1:** Retail sources and characteristics of white fish and eggs purchased in Australia for analysis of vitamin D_3_ and 25-hydroxyvitamin D_3_.

Location	Retailer Type	Description	Origin	Quantity Purchased (Fish) (kg)	Minimum Weight Per Dozen (Eggs) (g)	Free-Range Stocking Density (Eggs) (Hens/Hectare) [13]
Fish						
Adelaide	Supermarket	Blue grenadier hoki fillet, skinless	Not stated	2.1	-	
Brisbane	Supermarket	Hoki fillet, skinless, thawed, wild caught	New Zealand	2.0	-	
Melbourne	Seafood retailer	King dory fillet	Not stated	2.0	-	
Perth	Supermarket	Basa fillet, skinless, thawed	Vietnam	2.1	-	
Sydney	Seafood retailer	Barramundi fillet, skin-on, wild caught	Australia	2.4	-	
Eggs						
Adelaide	Supermarket	Cage	SA	-	700	-
	Supermarket	Free-range	SA	-	600	Not stated
Brisbane	Supermarket	Cage	QLD	-	700	-
	Supermarket	Free-range	QLD	-	700	10,000
Melbourne	Produce retailer	Cage	VIC	-	600	-
	Egg retailer	Free-range	VIC	-	800	130
Perth	Supermarket	Cage	WA	-	700	-
	Supermarket	Free-range	WA	-	700	1500
Sydney	Produce retailer	Cage	NSW	-	700	-
	Produce retailer	Free-range	NSW	-	660	10,000

SA: South Australia; QLD: Queensland; VIC: Victoria; WA: Western Australia; NSW: New South Wales.

**Table 2:** New data for moisture, fat, vitamin D_3_, 25-hydroxyvitamin D_3_ and vitamin D equivalents in white fish.

Purchase Location		Common Name (Origin), Systematic Name [21]	Moisture (g/100 g)	Fat (g/100 g)	Vitamin D_3_^(a)^ (μg/100 g)	25(OH)D_3_^(a)^ (μg/100 g)	VitDE ^(b)^ (μg/100 g)	True retention of Vitamin D_3_ (%) ^(c)^	True Retention of 25(OH)D_3_ (%) ^(d)^
Raw	Sydney ^(e)^	Barramundi, wild (Australia), *Lates calcarifer*	75.7	3.00	2.30	0.70	5.80	-	-
	Brisbane ^(e)^	Hoki, wild (New Zealand), *Macruronus* spp.	80.1	2.40	0.40	0.60	3.40	-	-
	Melbourne ^(e)^	King dory, *Cyttus traversi*	80.0	0.70	0.40	0.40	2.40	-	-
	Adelaide ^(e)^	Hoki, *Macruronus* spp.	84.8	1.50	0.10	0.30	1.60	-	-
	Perth ^(e)^	Basa, Pangasiidae	87.9	0.90	<0.1	0.30	1.50	-	-
Cooked	Sydney ^(e)^	Barramundi, wild, poached (Australia), *Lates calcarifer*	74.1	2.25	0.35	0.40	2.35	13	49
	Brisbane ^(e)^	Hoki, wild, poached (New Zealand), *Macruronus* spp.	78.1	2.25	0.20	0.45	2.45	40	60
	Melbourne ^(e)^	King dory, poached, *Cyttus traversi*	76.2	1.85	0.70	0.35	2.45	145	72
	Adelaide ^(e)^	Hoki, poached, *Macruronus* spp.	82.4	1.55	0.40	0.35	2.15	354	103
	Perth ^(e)^	Basa, poached, Pangasiidae	85.2	1.15	<0.1	0.60	3.00	NR	163

LOR = 0.2 μg/100 g for vitamin D_3_ and 0.1 μg/100 g for 25(OH)D_3_. Values for moisture, fat, vitamin D_3_ and 25(OH)D_3_ are the average of duplicate analyses. ^(a)^ Measured by high performance liquid chromatography with photodiode array; ^(b)^ VitDE = vitamin D_3_ + (5 × 25(OH)D_3_); ^(c)^ %True retention = (vitamin D_3_ per g cooked food × g of cooked food)/(vitamin D_3_ per g raw food × g of raw food) × 100; ^(d)^ %True retention = (25(OH)D_3_ per g cooked food × g of cooked food)/(25(OH)D_3_ per g raw food × g of raw food) × 100; ^(e)^ Average of duplicate analyses. 25(OH)D_3_, 25-hydroxyvitamin D_3_; VitDE, vitamin D equivalents; NR, not reported.

**Table 3:** New data for moisture, fat, vitamin D_3_, 25-hydroxyvitamin D_3_ and vitamin D equivalents in chicken eggs.

Purchase Location	Moisture (g/100 g)	Fat (g/100 g)	Vitamin D_3_^(a)^ (μg/100 g)	25(OH)D_3_^(a)^ (μg/100 g)	VitDE ^(b)^ (μg/100 g)	True Retention of Vitamin D_3_ (%) ^(c)^	True Retention of 25(OH)D_3_ (%) ^(d)^
Raw free-range							
Adelaide ^(e)^	76.2	9.6	1.7	0.7	5.0	-	-
Brisbane ^(e)^	77.1	9.0	1.5	1.5	9.0	-	-
Melbourne ^(e)^	76.1	9.7	2.4	0.7	5.7	-	-
Perth ^(e)^	76.1	8.4	0.6	0.5	3.1	-	-
Sydney ^(e)^	76.8	9.0	0.3	0.9	4.8	-	-
Average across cities	76.4	9.1	1.3	0.8	5.5	-	-
Raw cage							
Adelaide ^(e)^	75.4	10.7	0.8	1.3	7.0	-	-
Brisbane ^(e)^	76.2	9.6	0.7	0.8	4.7	-	-
Melbourne ^(e)^	76.4	9.7	0.5	1.3	7.0	-	-
Perth ^(e)^	75.7	9.4	0.6	0.6	3.6	-	-
Sydney ^(e)^	76.1	9.7	0.4	0.9	4.9	-	-
Average across cities	75.9	9.8	0.6	1.0	5.4	-	-
Hard-boiled free-range							
Adelaide ^(e)^	75.7	9.5	1.1	0.6	3.9	67	88
Brisbane ^(e)^	76.5	10.0	1.0	0.5	3.5	67	33
Melbourne ^(e)^	75.3	10.0	2.2	0.8	6.0	90	113
Perth ^(e)^	75.9	9.1	0.5	0.5	3.0	85	102
Sydney ^(e)^	76.4	9.4	0.3	0.8	4.3	103	92
Average across cities	75.9	9.6	1.0	0.6	4.1	80	75
Hard-boiled cage							
Adelaide ^(e)^	74.4	10.5	0.4	0.4	2.4	52	32
Brisbane ^(e)^	75.1	10.3	0.8	0.8	4.8	111	97
Melbourne ^(e)^	75.8	9.2	0.5	1.2	6.5	102	94
Perth ^(e)^	74.5	8.7	0.5	0.4	2.5	76	61
Sydney ^(e)^	76.1	10.1	0.5	1.0	5.5	126	112
Average across cities	75.2	9.7	0.5	0.8	4.3	89	77

LOR = 0.2 μg/100 g for vitamin D_3_ and 0.1 μg/100 g for 25(OH)D_3_. ^(a)^ Measured by high performance liquid chromatography with photodiode array; ^(b)^ VitDE = vitamin D_3_ + (5 × 25(OH)D_3_); ^(c)^ %True retention = (vitamin D_3_ per g cooked food × g of cooked food)/(vitamin D_3_ per g raw food × g of raw food) × 100; ^(d)^ %True retention = (25(OH)D_3_ per g cooked food × g of cooked food)/(25(OH)D_3_ per g raw food × g of raw food) × 100; ^(e)^ Average of duplicate analyses. 25(OH)D_3_, 25-hydroxyvitamin D_3_; VitDE, vitamin D equivalents.

**Table 4:** Internationally sourced data for moisture, fat, vitamin D_3_, 25-hydroxyvitamin D_3_ and vitamin D equivalents in white fish.

Country of Analysis	Common Name (Origin) and/or Systematic Name	Moisture (g/100 g)	Fat (g/100 g)	Vitamin D_3_ (μg/100 g)	25(OH)D_3_ (μg/100 g)	VitDE ^(a)^ (μg/100 g)	Method
Raw							
UK [18]	Cod	81.6	0.6	Trace	Trace	Trace	HPLC [24]
UK [18]	Haddock	81.7	0.4	NR	NR	Trace	HPLC [24]
UK [18]	Plaice	80.4	1.2	NR	NR	Trace	HPLC [24]
UK [18]	Sea bass	69.4	9.8	Trace	Trace	Trace	HPLC [24]
UK [18]	Lemon sole	82.7	0.7	NR	NR	Trace	HPLC [24]
USA [19]	Halibut (Atlantic and Pacific), *Hippoglossus hippoglossus* (L.) and *H. stenolepis* (Schmidt)	80.3	1.3	4.7	NR	4.7	HPLC/HPLC-LC/MS [22]
USA [19]	Ocean perch (Atlantic), *Sebastes marinus* (L.)	83.1	1.5	1.2	NR	1.2	HPLC/HPLC-LC/MS [22]
USA [19]	Cod (Pacific), *Gadus macrocephalus* Tilesius	84.0	0.4	0.5	NR	0.5	HPLC/HPLC-LC/MS [22]
USA [19]	Haddock, *Melanogrammus aeglefinus* (L.)	83.4	0.5	0.5	NR	0.5	HPLC/HPLC-LC/MS [22]
Cooked							
UK [18]	Cod, baked	76.9	0.5	Trace	Trace	Trace	HPLC [24]
UK [18]	Haddock, grilled	75.9	0.3	NR	NR	Trace	HPLC [24]
UK [18]	Haddock, steamed	78.2	0.6	NR	NR	Trace	HPLC [24]
UK [18]	Sea bass, baked	69.0	6.8	NR	NR	Trace	HPLC [24]
UK [18]	Lemon sole, grilled	76.8	0.6	NR	NR	Trace	HPLC [24]
USA [19]	Halibut (Atlantic and Pacific), dry heat, *Hippoglossus hippoglossus* (L.) and *H. stenolepis* (Schmidt)	76.1	1.6	5.8	NR	5.8	HPLC/HPLC-LC/MS [22]
USA [19]	Ocean perch (Atlantic), dry heat, *Sebastes marinus* (L.)	74.2	1.9	1.4	NR	1.4	HPLC/HPLC-LC/MS [22]
USA [19]	Cod (Pacific), dry heat, *Gadus macrocephalus* Tilesius	80.3	0.5	0.6	NR	0.6	HPLC/HPLC-LC/MS [22]
USA [19]	Haddock, dry heat, *Melanogrammus aeglefinus* (L.)	79.7	0.6	0.6	NR	0.6	HPLC/HPLC-LC/MS [22]

^(a)^ Calculated as: VitDE = vitamin D_3_ + (5 × 25(OH)D_3_). 25(OH)D_3_, 25-hydroxyvitamin D_3_; VitDE, vitamin D equivalents; HPLC, high performance liquid chromatography; LC/MS, liquid chromatography with mass spectrometry; NR, not reported.

**Table 5:** Internationally sourced data for moisture, fat, vitamin D_3_, 25-hydroxyvitamin D_3_ and vitamin D equivalents in eggs.

Country of Analysis	Type	Moisture (g/100 g)	Fat (g/100 g)	Vitamin D_3_ (μg/100 g)	25(OH)D_3_ (μg/100 g)	VitDE ^(a)^ (μg/100 g)	Method
Raw							
UK [18]	Average ^(b)^	76.8	9.0	2.5	0.18	3.2	HPLC [25]
USA [19]	Unspecified	76.2	9.5	2.0	NR	2.0	HPLC/HPLC-LC/MS [22]
The Netherlands [23]	Organic	75.9	9.5	1.5	0.1	1.5	HPLC [20]
The Netherlands [23]	Free range	76.2	9.1	0.9	0.1	0.9	HPLC [20]
The Netherlands [23]	Corn-fed	76.9	8.8	0.8	0.1	0.8	HPLC [20]
Cooked							
UK [18]	Average ^(b)^	75.4	9.6	2.30	0.18	3.2	HPLC [25]
USA [19]	Unspecified	74.6	10.6	2.2	NR	2.2	HPLC/HPLC-LC/MS [22]
The Netherlands [23]	Organic	75.8	9.5	2.1	0.2	2.1	HPLC [20]
The Netherlands [23]	Free range	76.6	8.5	1.5	0.2	1.5	HPLC [20]
The Netherlands [23]	Corn-fed	73.4	10.7	1.9	0.2	1.9	HPLC [20]

^(a)^ Calculated as: VitDE = vitamin D_3_ + (5 × 25(OH)D_3_); ^(b)^ Amalgamated sample of enriched cage, barn, free range and organic eggs. 25(OH)D_3_, 25-hydroxyvitamin D_3_; VitDE, vitamin D equivalents; HPLC, high performance liquid chromatography; LC/MS, liquid chromatography with mass spectrometry; NR, not reported.

## Abbreviations

The following abbreviations are used in this manuscript:

25(OH)D	25-hydroxyvitamin D
25(OH)D_3_	25-hydroxyvitamin D_3_
UV-B	Ultraviolet-B radiation
HPLC PDA	high performance liquid chromatography with photodiode array
VitDE	vitamin D equivalents
LOR	limit of reporting
LC-IT-MS	liquid chromatography-ion trap-mass spectrometry
